# Progress for Co-Incorporation of Polydopamine and Nanoparticles for Improving Membranes Performance

**DOI:** 10.3390/membranes12070675

**Published:** 2022-06-30

**Authors:** Nada Abounahia, Hazim Qiblawey, Syed Javaid Zaidi

**Affiliations:** 1 Desalination and Water Treatment, Center for Advanced Materials, Qatar University, Doha P.O. Box 2713, Qatar; na1507047@student.qu.edu.qa; 2 Department of Chemical Engineering, College of Engineering, Qatar University, Doha P.O. Box 2713, Qatar

**Keywords:** dopamine, polydopamine, mussel-inspired chemistry, nanoparticles, membranes

## Abstract

Incorporating polydopamine has become a viable method for membrane modification due to its universality and versatility. Fillers in their different categories have been confirmed as effective elements to improve the properties of membranes such as hydrophilicity, permeability, mechanical strength, and fouling resistance. Thus, this paper mainly highlights the recent studies that have been carried out using polydopamine and nanomaterial fillers simultaneously in modifying the performance of different membranes such as ultrafiltration, microfiltration, nanofiltration, reverse osmosis, and forward osmosis membranes according to the various modification methods. Graphene oxide nanoparticles have recently attracted a lot of attention among different nanoparticles used with polydopamine, due to their impressive characteristics impacts on enhancing membrane hydrophilicity, mechanical strength, and fouling resistance. Thus, the incorporation techniques of graphene oxide nanoparticles and polydopamine for enhancing membranes have been highlighted in this work. Moreover, different studies carried out on using polydopamine as a nanofiller for optimizing membrane performance have been discussed. Finally, perspectives, and possible paths of further research on mussel-inspired polydopamine and nanoparticles co-incorporation are stated according to the progress made in this field. It is anticipated that this review would provide benefits for the scientific community in designing a new generation of polymeric membranes for the treatment of different feed water and wastewater based on adhesive mussel inspired polydopamine polymer and nanomaterials combinations.

## 1. Introduction

Membrane technologies have evolved rapidly in recent decades in treating water and wastewater because of their significant equipment size reductions, reduced energy consumption, and inexpensive capital costs compared to conventional water treatment technologies. Micro-, ultra-, nanofiltration, and reverse osmosis have been considerably used in water treatment and desalination due to their efficient non-permeable performance and high-water recovery. However, high energy requirements, fouling, and scaling are still major concerns for these types of membranes that cannot be applied without pre-treatment stages [1]. Forward osmosis (FO) technology has recently received a lot of attention from a variety of industrial applications. This process can stand out as the most promising alternative for RO processes because of its high recovery rate, low energy demand, low fouling potential, and low pre-treatment requirements as compared to the other pressure-driven processes [2]. Nevertheless, FO technology has some drawbacks that restrict its ability, such as lower flux, internal concentration polarization (ICP), and reverse solute diffusion (RSD). As a result, several studies have been conducted in order to enhance different membrane permeability, selectivity, antifouling properties, and stability.

One of the most recent advancements to overcome the above-mentioned shortcomings in membrane technologies is the embedding of fillers into the membrane matrix through different modification techniques, which effectively changes the resulting membrane properties, structure, and predictable separation performance. In addition, these fillers have successfully improved different membranes fluxes and mechanical strength. Common inorganic fillers include elemental oxides (e.g., ZnO, SiO_2_) [3], nanoparticles (e.g., carbon nanotubes (CNT), TiO_2_, halloysite nanotubes (HNTs)) [4,5], graphene oxide (GO) [6,7], metal-organic frameworks (MOF) and metal nanoparticles (Ag) [8,9]. The most well-known hydrophilic nanomaterial that has lately become widely used is graphene oxide (GO) nanoparticles, which have attracted a lot of attention as a nanofiller and shown promising results in several studies because of their one-of-a-kind characteristics such as (1) a high specific surface area that improves contact with the polymeric support layer; (2) high chemical-mechanical stability; and (3) excellent hydrophilicity due to the presence of oxygenous functional hydrophilic groups such as hydroxyl, carboxyl, and carbonyl groups [10,11]. However, issues such as nanomaterial agglomeration, poor dispersion, and releasing some of these materials from the membrane matrix can compromise the membrane’s structural stability and solute selectivity. Therefore, several studies have preferred to adopt materials inspired by the adhesive secretions of mussels such as dopamine (DA), which are rich in abundant functional groups such as amine, catechol, and imine [12]. Dopamine is mostly incorporated to impart membranes anti-fouling and mechanical properties [12]. However, recently it has been integrated with nanomaterials in order to strengthen their stability, binding kinetics, and reduce their defects in the membrane matrix by different mechanisms for modifying the membranes. Therefore, it has been used as an interlayer for post functionalization before embedding the nanomaterials [13], co-deposited simultaneously with nanomaterials on the membrane’s surface [14], blended with nanomaterials into the membrane polymer matrix [15], and incorporated with nanomaterials into a polyamide selective layer [15]. In another way, it has been widely used to functionalize a variety of nanomaterials (ultrathin imprinted polydopamine (PDA) films on the surface of nanomaterials) before embedding them into the membrane by either simple coating and deposition or phase inversion or during interfacial polymerization methods [16]. However, no comprehensive review of the use of polydopamine and nanomaterials in improving water purification membranes has been conducted. Therefore, this work aims to review all the previously mentioned membrane modification methods based on PDA and nanomaterials combinations for modifying UF, MF, NF, RO, and FO membranes. This work will also highlight the co-incorporation techniques employed in the previous studies between GO nanoparticles and PDA in enhancing the membranes. The use of PDA as nanoparticles for membrane modification was briefly mentioned at the end of the study. We believe that this study will help the researchers in this field by opening new possibilities in designing a new generation of polymeric membranes for the treatment of different feed waters and wastewater based on adhesive PDA polymer and NPs combinations.

## 2. Polydopamine

**Figure 1:**
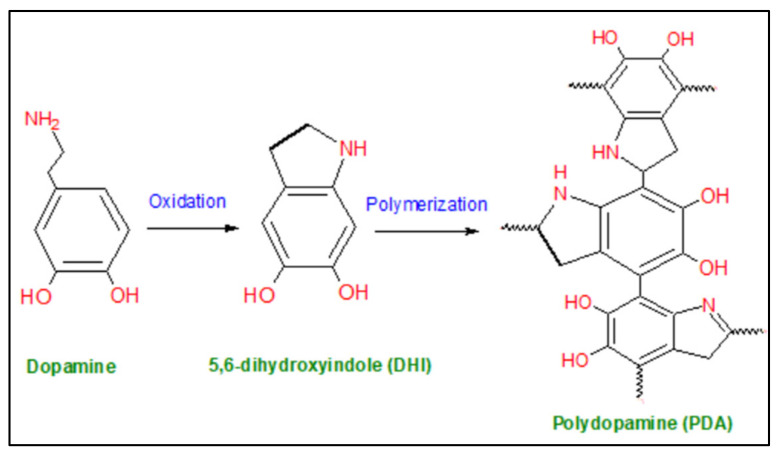
Polydopamine (PDA) formation.

Recently, membrane enhancement employing materials inspired by the adhesive secretions of mussels such as dopamine (DA) with molecular formula C_8_H_11_NO_2_ has attracted great scientific interest. Dopamine is known as a bio-adhesive or mussel inspired bio-glue that is utilized to modify the surface of various inorganic and organic substances through self-polymerization on the materials’ surface, forming thin polydopamine (PDA) layers as shown in Figure 1 [16,17]. Dopamine is a molecule that has a high rate of reactivity with the highest oxidation potential among catechol amines. PDA possesses catechol groups, primary amines, and secondary amines that can easily oxidize to create reactive quinone, which combines with a variety of functional groups, including amines and thiols, to form covalently grafted functional layers via Michael addition or Schiff base reaction [16,18]. PDA is insoluble in water and organic solvents, which makes it a suitable candidate for membrane modification [12]. Dopamine-based modification has been proven as an efficient modifier that has a significant impact on membrane properties such as chemical properties, hydrophilicity, morphology, and mechanical strength. Thin polymeric films of polydopamine (PDA) are usually prepared via the self-polymerization (oxidation) of dopamine monomer in weak alkaline conditions (pH = 8.5) to stimulate the oxidation of a catechol structure into quinones and facilitate the crosslinking reaction [19]. Due to the advantages of PDA mentioned above, numerous researchers have exploited PDA as a surface modifier for different membranes or as an interface layer for post-modification that permits other functional materials, such as nanoparticles, or polymers, or oligomers, to further modify the membrane [20].

## 3. Modification Techniques of (UF, MF, NF, and RO) Membranes through Co-Incorporation of PDA and NPs

### 3.1. Two-Step Modifications (PDA-Based Post-Functionalization)

**Figure 2:**
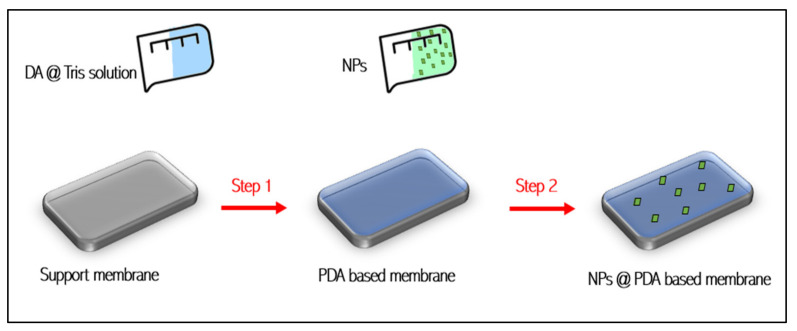
Two-step modification technique.

The two-step modification technique is the introduction of functional molecules (such as thiols and amines) onto the formed PDA layer where a Michael addition reaction and/or Schiff base reaction between the grafted functional molecules and quinone functional groups of the PDA layer occurs [12]. This method has been investigated by several studies due to its ability to increase membrane antifouling properties. Polydopamine is capable of holding the nanomaterials on specific surfaces, where these materials are attached to the surface via a chemical reaction/immobilization as shown in Figure 2. By this concept, PDA can create a hydrophilic layer and increase membrane stability for long-term operation by controlling the release of NPs from the PDA-coated membrane matrix due to the presence of catechol groups in PDA that have a high affinity toward transition metals such as silver [13]. In addition, coating the membrane surface with DA can lead to the formation of noble metal nanoparticles on the surface as well as on the pore walls of the membrane without the need for a reducing agent in the case of using anions such as [AuCl_4_]^-^ anions, which can be reduced to gold nanoparticles [21]. Furthermore, in another study, PDA was utilized to improve the adhesion and stability of titanium dioxide (TiO_2_) nanoparticles on a polyethersulfone (PES) ultrafiltration membrane [20]. The modification procedure was carried out by dipping the membrane substrate into PDA solution followed by TiO_2_ NPs self-assembly deposition over the PDA-modified PES membrane without carrying out PA crosslinking reaction [20]. The resulting membrane performed admirably in terms of BSA rejection, achieving 82% but at about 50% flux reduction. Thus, besides increasing membrane hydraulic resistance, in the case of NPs overloading, this method could increase the surface pores blocking of the membranes due to the PDA layer that causes many more NPs to attach and cluster [20]. This results in a higher flux reduction due to the disconnection of the link between the macrovoids [13]. However, the co-deposition of zwitterionic polymer and polydopamine (PDA) onto the membrane surface followed by embedding silver nanoparticles (Ag NPs) as a second step can reduce NPs agglomeration [22]. Moreover, zwitterionic polymers could repair the PDA layer, which had a high capacity to disperse NPs on the surface and thus extend the bactericidal period. In another experimental work [23], polypropylene microfiltration membrane (PPMM) with excellent antifouling and hydrophilic surfaces has been achieved by co-depositing PDA/PEI as an intermediate layer followed by embedding TiO_2_ nanoparticles (NPs) as a second step through a sol-gel process. PEI enables the membrane surface and inner pores to be well coated. While PDA/PEI facilitates the introduction of TiO_2_ NPs onto PPMMs conveniently. The modified membrane showed a significant increase in water flux (J_w_ = 5720 L m^−2^ h^−1^ (LMH)) compared to the pristine membrane (J_w_ = 605 LMH) under 0.1 MPa [23]. Despite the fact that the two-step modification needs a long time for grafting processes, it ensured more efficient grafting of subsequent NPs on the membrane’s top layer.

### 3.2. One-Step Modification Method (Dopamine-Assisted Co-Deposition)

**Figure 3:**
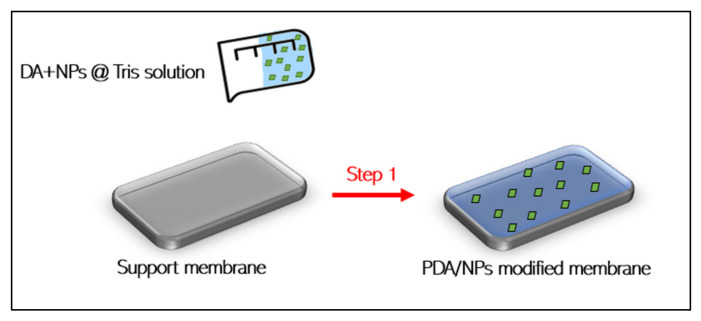
One-step modification technique.

PDA through self-polymerization reaction of dopamine in the air is considered a time-consuming process that dominated the non-covalent interaction and where the crosslinking rate of dopamine is controlled by the oxidation degree of dopamine [24]. Furthermore, after a long deposition period, non-covalent interactions in solutions such as acidic or alkaline aqueous solutions and polar organic solvents cause PDA oligomers to cluster, which can block membrane pores and reduce water permeability due to the resulting unstable coating [24]. To shorten the long self-polymerization duration of dopamine and PDA aggregation due to the long deposition issues, a new technique has been proposed: “one-step modification based on dopamine self-polymerization”. It was confirmed that this technique can speed up the deposition process by increasing the covalent binding of dopamine, resulting in a stable and uniform PDA coating, paving the way for the development of mussel-inspired chemistry [24]. Moreover, it can reduce the self-aggregation of PDA to form particles and then promote the homogeneous polymerization and deposition of dopamine [25]. One-step modification relies on mixing nanomaterials directly with dopamine in the deposition solution, forming covalent crosslinking or non-covalent interaction and contributes to the formation of co-depositing surface coatings as illustrated in Figure 3. Co-depositing PDA over membrane surfaces with organic or inorganic nanoparticles can increase the filtration capability of the membrane, and this method has been extensively studied by researchers due to its impact on the functionalization/modification of nanomaterials and at the same time boosting the oxidation of dopamine. Some of the multifunctional nanomaterials that have been co-deposited with PDA are TiO_2_ nanoparticles [14], Cu NPs [26], SiO_2_ NPs [27], gold nanoparticles (GNPs) [28], and lead (Pd) NPs [29], etc., which are all summarized in Table 1. Co-depositing of these nanomaterials with PDA deposition solution onto the membrane surface has resulted in significant improvements in various membranes performances including increased membranes hydrophilicity [13,30], membranes salt and dye rejection [26,28,31,32,33], membranes stability and mechanical strength [27,33]. As a result, all of these co-depositing membranes demonstrated a competitive and practical solution for long-term management of highly saline wastewaters, such as textile wastewater. Another advantage of co-depositing PDA with NPs can be noticed in the case of using more than one nanomaterial type, in this case, co-depositing PDA with these hydride nanomaterial combinations can enhance the crosslinking between them, leading to a stronger adhesion on the membrane support layer [34].

### 3.3. Functionalization of NPs by PDA

The presence of abundant functional groups on the PDA surface increases its efficiency for the functionalization/modification of several nanomaterials such as multi-walled carbon nanotubes, Ag NPs, SiO_2_, TiO_2_, and GO NPs. Chemical bonding (Michael addition or Schiff base reactions) or physical bonding (π–π stacking or hydrogen bond) is used by these groups to introduce functional molecules onto nanoparticles [35]. Nanoparticles that have been modified have been widely used in medical applications such as drugs carriers and biosensors [35,36,37,38,39,40,41]. It is used for environmentally friendly catalyst preparation [42,43,44,45] and detection and degradation of pesticides [46] too. Moreover, it can be used as nano-adsorbents for water remediation [47] and as modifiers for water purification membranes [16], as will be illustrated in the following sections.

**Figure 4:**
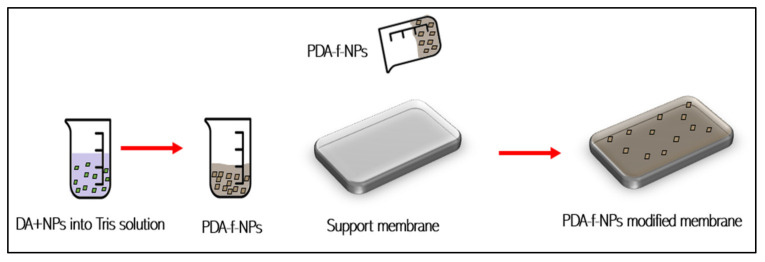
Simple deposition modification method using PDA-f-NPs.

PDA post-treatment of NPs prior to use as an additive in polymers is accomplished by dispersing them in a dopamine tris(hydroxymethyl) aminomethane (Tris) solution, where oxidative self-polymerization of dopamine occurs on the surface of the NMs, as shown in Figure 4. This technique increases nanoparticles’ binding on the membrane surface and achieves uniform dispersion for constructing membranes with stable, long-lasting high performance without significantly changing the morphology of the nanoparticles before and after functionalizing or altering their basic chemical structure [15,48]. These functionalized or modified DA-NPs can be incorporated into membranes through simple dip coating, vacuum filtration deposition, phase inversion, or they can be introduced into the PA layer through the interfacial polymerization (IP) method as summarized in Table 2.

#### 3.3.1. PDA-f-NPs Coating and Deposition Modification Methods

A simple, practical, and facile coating technique of a variety of membranes was proposed by a number of studies using PDA-f-NPs. A single step in situ dip coating of the hydrophilic layer of PDA-f-TiO_2_ is used to modify UF-PES membranes [16]. The modified membrane with a small pore size improved the membrane selectivity with improved hydophilicity and permeate flux. Moreover, when compared to the pristine one, it had better antifouling and antibacterial capabilities. The coating layer was also found to be stable after a long period of use. However, inducing nanomaterials (NMs) as an interlayer between the substrate membrane and the PA skin layer can reduce the incorporation of NMs and avoid their wastage during the TFN preparation. Therefore, dopamine has been widely used to minimize NMs agglomeration, which enhanced their dispersion in aqueous solution and consolidated the surface interactions between the PA matrix and NMs. A unique hybrid nanostructure (HNS) has been created through using metal/metal oxide (M/MO) nanoparticles (Ag/Al_2_O_3_, Fe_2_O_3_, and TiO_2_) which were loaded on the surface of carbon nanotubes (CNTs) [49]. These HNS were then coated with a thin polymeric film of PDA and deposited on a PES substrate membrane, followed by an interfacial polymerization (IP) procedure that resulted in a thin layer of polyamide (PA) above the intermediate layer. When compared to the thin film composite TFC membrane, the manufactured TFN-NF membranes performed better in terms of permeability properties [49]. On the other hand, issues such as agglomeration of NPs inside the porous media and the large quantity of NPs required to provide uniform distribution throughout the membrane porous structure could limit the use of NPs. Thus, these disadvantages can be overcome by introducing NPs into the active layer (top surface) of the membrane. For example, incorporating copper-MOF (Cu-MOF) nanoparticles with the PDA for active layer surface coating of PES-NF membranes resulted in high membrane permeability, high surface hydrophilicity, and high dye rejection [50]. The coating method was performed by utilizing two different simple techniques static: (dip-coating) and dynamic (filtration-assisted) fabrication processes.

#### 3.3.2. PDA-f-NPs Blending Modification Method

**Figure 5:**
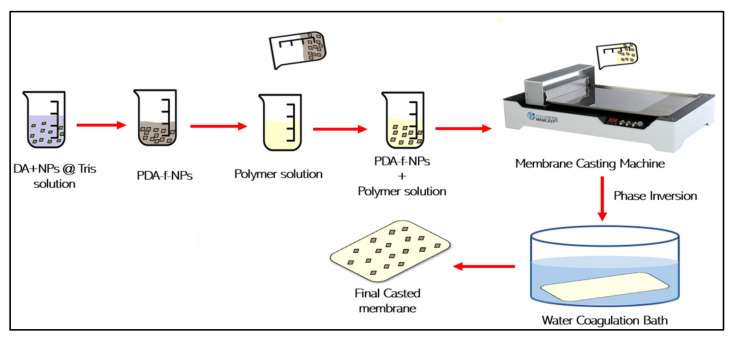
Blending (phase inversion) modification method using PDA-f-NPs.

The blending modification technique is based on blending PDA-f-NPs with membrane polymer matrix/film (casting solution) followed by the phase inversion method for preparing the membrane as demonstrated in Figure 5. DA-modified NPs can lead to the formation of homogeneous dispersed nanocomposite membranes even at high concentrations of nanoparticles and improve the interfacial compatibility between the nanofillers and the polymer matrixes, unlike unmodified NPs, which show high exclusion from the membrane matrix and indicate a low nanoparticle–polymer interaction [51,52]. PDA-f-TiO_2_ nanohybrid NPs have been doped into PSf matrix and PVDF matrix via the phase inversion method in two different studies [53,54]. The PSf membrane achieved its optimal membrane filtration properties by loading 0.8 wt% PDA-f-TiO_2_, indicating a remarkable self-cleansing property and correct long-term performance steadiness [53], whereas PDA-f-TiO_2_/PVDF improved membrane antifouling property and increased membrane flux [54]. Another PDA-coated nanomaterial which has been used to enhance the PSf-UF membrane by a phase inversion technique is multiwalled carbon nanotubes (MWNTs) [55]. The PDA-MWNT/PSf maintained a good rejection performance (99.88%) with high membrane permeability up to 50% for the optimum dose of 0.1 wt% of PDA-MWNTs. As well, the prepared membrane showed higher mechanical strength and long-term stability for ultrafiltration operation [55]. In addition, using the non-solvent induced phase separation (NIPS) method, new polydopamine (PDA)-coated ZnFe_2_O_4_ nanocomposites were incorporated into the PES casting solution [56]. The pure water flux, humic acid (HA) removal efficiency, and separation of the oil/water emulsion for the developed hybrid membrane with 4 wt% PDA@ZnFe_2_O_4_ reached ~687 LMH, 94%, and 96%, respectively [56]. Another dopamine-functionalized NP is dopamine (DA)-coated silica nanoparticles, which have been blended with PAN solution for preparing hydrophilic UF membranes. In this, no NPs agglomeration has been observed during long-term storage due to the presence of DA. The prepared PAN–SiO_2_-DA membrane by solution casting showed an enhancement in membrane filtration and rejection performance for bovine serum albumin (BSA) protein and Congo red dye [51]. In recent studies, new NPs have been prepared using zwitterionic monomers such as sulfobetaine methacrylate (SBMA) and DA to prepare P(DA-SBMA) nanoparticles [57,58]. Wet phase inversion is used to embed the new P(DA-SBMA) nanoparticles into a cellulose acetate mixed matrix [57]. The modified CA membrane showed optimal water flux of 583.64 LMH with enhanced reversible fouling by 11.10% and achieved high separation efficiencies for treating different types of oily wastewater (95–99%).

#### 3.3.3. PDA-f-NPs during IP Modification Method

**Figure 6:**
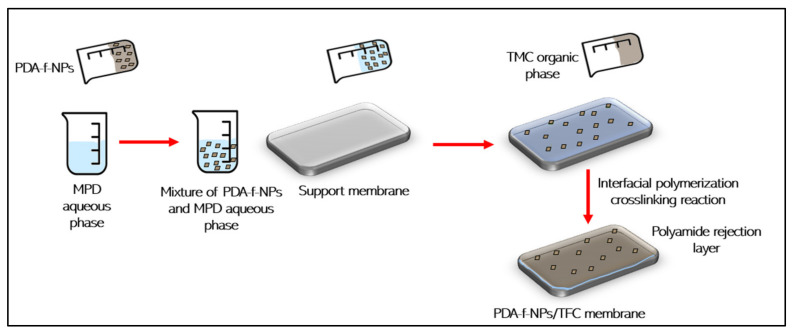
Embedding PDA-f-NPs into MPD aqueous phase followed by creating PA rejection layer by interfacial polymerization crosslinking method.

Another advanced method for incorporating PDA-f-NPs into TFN membranes is by embedding these functionalized NPs into the crosslinked ultrathin barrier layer of a polyamide (PA) TFC membrane, as shown in Figure 6. By this method, some morphological changes of the PA-TFC membrane can be observed. The PA-TFC membrane was found to have a relatively rougher crumbled structure. Meanwhile, introducing modified NPs by PDA into the PA selective layer has made the crumpled structure of the TFC membrane smoother as well as the tufts become shorter and narrower [48]. A modified hydrophilic zeolitic imidazolate framework-8 (ZIF-8) nanoparticles by polydopamine modification were highly dispersed in a well-mixed aqueous solution containing 2 wt% piperazine (PIP), 2 wt% triethylamine (TEA), 4.6 wt% camphorsulfonic acid (2CSA), and 0.01 wt% PDA-ZIF-8 nanoparticles for enhancing PA layer formation onto PSf membrane surface [59]. The TFN membrane that resulted in a negatively charged surface has increased water permeability without sacrificing selectivity and ensured that multivalent anions and dyes were effectively rejected [59]. Whereas, PDA-coated SiNPs (PDA-f-SiNPs) were utilized for preparing PSf thin-film nanocomposite membranes by adding the modified PDA-f-NPs to the organic phase during the interfacial polymerization process [60]. The PDA coating creates more water channels at the interface between NPs and the PA matrix. The PA thickness layer of the modified membrane with PDA-f-SiNPs was thinner because PDA-f-SiNPs interfered with the reaction between PIP and TMC to a higher extent, resulting in a slower reaction rate and, as a result, a thinner layer [60]. Hence, water resistance was reduced and water flux increased by 91.1%, while salt rejections for Na_2_SO_4_, MgSO_4_, MgCl_2_ and NaCl were 97%, 94%, 68%, and 35%, respectively [60]. The TFN membrane also exhibited high antifouling and stable performance. In another experimental work, P(DA-SBMA) nanoparticles were incorporated into the PA layer by dispersing in the TMC organic phase [58]. The PSf TFN membrane prepared via the IP process displayed good fouling resistance, yielding a high flux recovery rate (99.53%) even after exposure to BSA foulant [58]. With the same concept as the above-stated study, ZIF-8@PDA nanoparticles have been embedded into the PA layer via the IP process to modify the commercial PSf-UF (20 kDa) membrane [61]. After modification, the results manifested a promising hydrophilic and smooth membrane with high stability performance under the fouling test.

## 4. Modification Techniques of FO Membranes through Co-Incorporation of PDA and NPs

Despite the fact that FO is a promising technology with low fouling potential, low energy consumption, and minimal infrastructure needs, the flux of FO is still inferior to RO at similar theoretical applied pressures [62]. Several studies using mussel-inspired PDA polymer reported impressive high performance of various FO membranes. The modification was based on using PDA as free-standing or combined with different nanomaterials, which will be discussed below.

### 4.1. PDA-Based Modification

Utilizing PDA bio-inspired polymer for enhancing forward osmosis membrane has been studied by some researchers as shown in Table 3, and its deposition process into FO membranes has been done through different techniques such as dip coating, vacuum filtration deposition, one-step co-deposition, and interfacial polymerization. Different RO membrane support layers such as BW30 and SW30-XLE were enhanced through the coating of their polysulfone (PSf) support layers by DA [63]. The enhanced membrane exhibited a high-water flux with low ICP under FO test conditions and good desalination performance with a 2 M NH_3_–CO_2_ draw solution and a 0.25 M NaCl feed [63]. In another study, the prepared PSf membrane substrate through the casting method was modified by PDA coating prior to the IP process in order to enhance the stability between the PA active layer and the substrate membrane [64]. Consequently, the enhanced membrane showed higher water flux (24 LMH) and salt rejection properties (85%) compared to the TFC-PSf membrane with 7.5 LMH water flux and 80% salt rejection [64]. Both membranes were tested using deionized water as a feed solution and 2 M NaCl as a draw solution and operated in pressure retarded osmosis (PRO) mode where the active layer faced the draw side (AL-DS) [64]. It was also indicated that short PDA coating times on membrane substrates could decrease the thickness of the PA layer and increase salt rejection. Furthermore, a PVC membrane synthesized via phase inversion was modified via PDA coating (1–3 h) as a mid-layer before PA active layer preparation [65]. The resultant PDA-TFC FO membrane displayed high water flux (18.90 LMH) in FO mode and lower reverse solute flux (RSF) (3.35 g m^−2^ h^−1^ (gMH)) using DI water as FS and 1 M NaCl as DS [65]. However, a new TFC FO membrane was fabricated through a simple uniform dip coating of pristine polyethylene (PE) support into dopamine solution for 8 h, followed by forming a selective PA layer on top of hydrophilic polydopamine (PDA)-modified polyethylene (DPE) support via the IP technique [66]. In comparison to other lab-scale and commercial membranes, the resulting DPE-TFC membrane had a greater FO water flow and a lower specific salt flux, as well as outstanding long-term stability and mechanical resilience. In order to increase the salt rejection of one of the most commonly used FO membranes, which is the cellulose acetate (CA) membrane, it is recommended to coat the membrane with PVA before coating with PDA. In another study, CA membrane has been modified via the phase inversion method by using PVA and PDA coating techniques [67]. PVA was cross-linked onto the surface of CA membranes before being coated with PDA using a fast deposition process. The improved membrane demonstrated higher hydrophilicity and displayed 16.72 LMH and 0.14 mMH osmotic water flux, and reverse solute flux, in FO tests utilizing DI water and 2 M NaCl as feed and draw solutions respectively, with the active layer facing the feed solution [67].

However, exposing the rejection layer of FO membranes surface to the PDA coating reveals another level of enhancement, in which a few studies have applied this concept to FO membranes aiming to increase their antifouling behavior. An experimental study showed that the PDA-coated commercial membrane TFC with a coating duration of 0.5 h had a better antifouling performance with low surface roughness during alginate fouling as well as a significant improvement in membrane hydrophilicity [71]. A PK-TFC membrane was fabricated via the phase inversion method of the PK support layer followed by IP reaction between the aqueous MPD phase and organic TMC phase to prepare the PA rejection layer [68]. The prepared PK-TFC membrane was finally modified by single step co-deposition of PDA and MPC-co-AEMA polyamphoteric polymer atop the TFC PA active layer, forming a PK-TFC-PDA/MPC FO membrane with high fouling-resistance properties during protein-containing wastewater and high concentration oily emulsion treatment [68].

Incorporation of PDA alone into one of the PA rejection layer phases during the IP method is considered one of the recent novel techniques that has been used to fabricate an FO membrane with high performance. However, researchers preferentially introduced PDA into the MPD aqueous phase solution rather than the TMC organic phase to preferentially decrease the PA layer cross-linking degree and increase the membrane hydrophilicity, resulting in a higher driving force for water molecules during the FO process [69]. Mixed cellulose ester (MCE) substrate was modified based on DA-incorporated TFC via introducing DA into the MPD aqueous phase, which showed a good enhancement in cross-linking degree between TMC and MPD-DA during the IP process [69]. As well, under FO experiment test conditions using deionized water and 1 M NaCl as feed and draw solutions, respectively, the modified membrane demonstrated a high water flux of 50.5 LMH, which was enhanced three fold over the traditional TFC (TMC/MPD) membrane with a comparable RSF of 8.19 gMH, while maintaining NaCl rejection over 92% in PRO mode [69].

Dopamine concentrations combined with MPD in the aqueous phase can have an undesirable impact on the characteristics and performance of FO membranes. Some studies have been directed recently to study the relationship between the DA self-polymerization concentration in the aqueous phase of the PA layer and FO membrane performance, using casted polysulfone substrates [72]. It has been reported that decreasing the concentration of DA in the aqueous phase can reduce self-polymerization and PDA formation, as well as limit the polymerization reactions between MPD and TMC monomers [72]. This will lead to a more compact, denser structure, lower surface roughness, a more hydrophilic surface, and a thinner PA active layer, which are highly desirable for achieving high selectivity and high antifouling properties. In contrast, increasing DA concentration in the aqueous phase causes excessive PDA particle aggregation and less attractive force between MPD monomers and PDA particles, which leads to a loosely packed, rougher structure, and a thick PA layer that can sacrifice the selectivity. In another study, dopamine was used as a sole monomer in the aqueous phase to react with the TMC organic phase, creating an active layer through self-polymerization of DA and interfacial polymerization of TMC in FO membrane synthesis [73]. When the membrane was subjected to a chloride resistance test, the newly produced active layer on top of the polysulfone substrate with ester bonds made by DA/TMC was considerably more stable than the amide bonds of the PA layer [73].

### 4.2. Combination of PDA and NPs-Based Modification

PDA polymer can play an important role in bounding NPs onto FO-TFC membrane in order to save its PA layer from chlorination. This was demonstrated in a study in which a PSf support was prepared using the phase inversion method and a PA layer was created using the IP technique [70]. The prepared TFC was coated by PDA self-polymerization, and finally, the PDA-TFC membrane was immersed in the Mg_3_Al-CO_3_ LDH nanoparticle suspension for 1 h. The fabricated membrane indicated a promising anti-fouling capability with a high chlorine-resistant time [70]. For further enhancement during incorporation of DA into one of the PA layer’s phases, doping nanomaterials at the same time into one of the phases has recently attracted great attention due to the increased number of water channels in the PA layer and the huge modification in TFC membrane separation performance achieved by this technique. A designed double-layer polyacrylonitrile (PAN) ultrafiltration membrane as a support layer has been modified by pouring PDA/MPD aqueous solution at the top followed by dispersing metal organic frameworks (MOF)/TMC organic solution by the IP process, forming a thin film nanocomposite (PDA/MOF-TFN) forward osmosis (FO) membrane [15]. The results revealed that the novel PDA/MOF-TFN membrane can increase the water flux by 30%, and decrease the RSF by 44% compared to the TFC membrane, while achieving a high removal rate of 94~99.2% for Ni^2+^, Cd^2+^, and Pb^2+^ in heavy metal wastewater treatment [15].

For FO membrane enhancement, a few studies have implemented dopamine-functionalized nanomaterials. Both sides of the polyethersulfone (PES) microfiltration (MF) membrane have been modified through the depositing of polydopamine-functionalized SWCNTs (PDA-SWCNTs) using vacuum filtration and spraying techniques [74]. The findings showed that the TFC-modified membrane (sandwich-like SWCNTs-coated support) had an excellent water flux value of 35.7 LMH and a low reverse salt flux of 1.42 gMH when tested in PRO mode (AL-DS) using 1 M NaCl and DI water as a draw and a feed solution, respectively. It also had superior antifouling properties, with a relative fouling degree (RFD) of 19.05 in the cross-flow test and 8.4% in the BSA adsorption test [74]. Furthermore, another study used PDA to modify the zeolitic imidazolate framework (ZIF-8) to improve ZIF-8 dispersion in water [75]. The ZIF-8@PDA was incorporated into the PEI aqueous solution required for preparing the selective layer on top of the polyethersulfone ultrafiltration membrane. The membrane was then contacted with TMC organic solution where the IP reaction started taking place [75]. The use of ZIF-8@PDA increased water permeability without losing selectivity, resulting in a high separation efficiency for heavy metal ion removal by the FO process.

## 5. Graphene Oxide (GO) Nanoparticles

Graphene is a two-dimensional substance made from natural graphite (Gr). It is made up of sp2 hybridized carbon atoms that are arranged in a honeycomb pattern. Graphene oxide (GO), which is made by oxidizing graphite, is one of the most intensively studied graphene-based compounds [76]. Because of its unique properties, graphene oxide (GO) has been demonstrated as a high potential emerging nano-building material for the fabrication of novel separation membranes. In comparison to other carbon-based materials, GO is more cost-effective [77]. The high concentration of oxygenous functional groups such as epoxy, hydroxyl, carbonyl, and carboxylic groups in GO boosts its solubility in water and in a variety of solvents [78,79]. As a result, GO film can be deposited into any substrate using the most appropriate approach. Moreover, the presence of these groups has enhanced the GO hydrophilicity, which consequently increases the water permeability through GO incorporated membranes due to the creation of hydrogen bonds between the membrane surface and water. Embedding GO nanoparticles into the membrane matrix has improved fouling resistance due to the carbon-based affinity of GO carbon particles, which absorb fouling agents and increase membrane rejection of dyes, oil, and salt while reducing surface roughness [80]. GO has a high thermal stability and a high specific surface area of about 890 m^2^g^−1^that enhances interaction with the polymeric support layer and high mechanical strength [81]. The Hummers’ method, first reported in 1958, is currently the most widely utilized method for GO synthesis. For graphite oxidation, potassium permanganate (KMnO_4_), sulfuric acid (H_2_SO_4_), and sodium nitrate (NaNO_3_) are utilized [76]. According to that, several studies have focused on the simultaneous use of GO and PDA in improving membrane separation performance using either unfunctionalized or functionalized GO NPs, as shown in Table 4 and Table 5. The reason behind this high interest in PDA and GO NPs combination is the potential to combine the beneficial features of PDA and GO, resulting in highly stable reduced GO particles with extraordinary hydrophilicity and dispersity in different organic solvents as compared to pristine GO. Amines have also been discovered to improve GO NPs conductivity, antifouling and antibacterial properties, surface area, adsorption capacity, and mechanical and thermal stability [82,83].

### 5.1. Unfunctionalized GO NPs

For the manufacture of stable GO membranes, a pre-modification technique for the membrane support surface employing PDA coating is proposed. The addition of polydopamine aids in the binding of GO nanosheets to the support surface. By coating polyether sulfone support layer surfaces with PDA and then depositing GO laminates to form the separation layer, a versatile adhesive platform was created [84]. The new modified NF membrane with high structural stability achieved 85 LMH/bar water permeability and retained Methyl Orange, Orange G, and Congo Red at 69%, 95%, and 100%, respectively [84]. High desalination performances were noted for GO/PDA-modified supports. PDA/GO can provide an efficient membrane for treating oily wastewater like oil/water emulsions. High oil rejection of over 91% has been successfully achieved by using highly stable hydrophilic GO/PDA/MCEM, which is prepared by a simple vacuum filtration method on the PDA-functionalized mixed cellulose ester membrane (MCEM) [85]. The same vacuum filtration method was followed to form a dense and stable GO layer onto a PDA-modified-alumina (Al_2_O_3_) support surface, leading to a high ion rejection of over 99.7%, making it promising for seawater desalination on a large-scale [86]. The prepared modified electrospun Poly(arylene ether nitrile) (PEN) nanofibrous mats (supporting layer) demonstrated remarkable antifouling performance for various oil/water emulsions and excellent reusability, which were synthesized by controlled assembly of HNTs intercalated GO (skin layer) through vacuum filtration onto the surface of electrospun PEN nanofibrous mats and further mussel-inspired PDA coating [87]. In another study, the same electrospun PEN membrane and modification technique for forming a hydrophilic GO-PDA skin layer were prepared, showing that hot-pressing electrospun PEN before modification could provide high water flux and stability (including thermal stability and high mechanical strength) [88]. Moreover, the use of SiO_2_-intercalated RGO-based ultrathin laminar films on the PVDF support layer via facile vacuum filtration approach followed by introducing DA demonstrated high stability, wettability, and antifouling ability with great promise performance in oil–water emulsion and dye wastewater treatment [89]. Up to now, vacuum filtration has been the most commonly used GO deposition technique to form a uniform GO skin layer onto the membrane substrate surface. However, the drop-casting method based on the evaporation process has also achieved uniform and flattened reduced graphene oxide films on polydopamine-modified PET substrates [90]. Additionally, antifouling properties for PS support membrane were improved by depositing GO on the surface of a dopamine-modified polysulfone ultrafiltration membrane through a layer-by-layer (LBL) self-assembly method, achieving superior NF performance with about a 98% rejection rate of methyl blue [91]. Another antifouling test was conducted using a sodium alginate fouling test for the modified PSF/PDA/aGO membrane in which aGO stands for activated GO (aGO) containing amine-reactive esters [92]. The PSF/PDA/aGO membrane showed a 54% lower fouling rate than the unmodified PSf, and demonstrated stability for 48 h of operation and interval cleanings using sodium hydroxide (NaOH) solutions. On the other hand, coating a binding agent such as polydopamine (PDA) and graphene oxide (GO) over the membrane rejection layer can strengthen the membrane anti-fouling properties [93]. For example, PDA-GO printed NF membranes (NF90) were constructed via an inkjet printing technique [93]. The DA solution was printed on the membrane surface first, followed by the GO solution, and finally, the tris(hydro-xymethyl)aminomethane hydrochloride (tris-HCl) buffer solution was printed as the final layer on the membrane surface to increase the DA self-polymerization rate [93]. PDA served as a strong binding agent between the GO and PA active layers, ensuring chemical and mechanical stability of the composite membrane. The results showed a higher salt rejection performance compared to the control polymeric NF membrane but with a slightly lower permeate flux.

In the FO system, modifying CTA-ES membrane with rGO then dipping it into dopamine solution increased its water flux from (23.6 LMH) for rGO-membrane to (34.0 LMH–36.18 LMH) for rGO-PDA membrane with greatly reduced reverse solute flux, indicating PDA’s ability to reduce surface hydrophobicity and facilitate water entry into the nanochannels [94,95]. However, the deposition of PDA with other nanoparticles such as silver nanoparticles (nAg) can increase rGO membrane biofouling resistance and ion rejection in the FO system [94]. Nevertheless, silver release from these membranes is a critical problem that causes water permeation decline [94].

### 5.2. Dopamine-Functionalized GO NPs

Graphene oxide can strongly react with other functional groups due to the presence of oxygen-containing groups. In this way, GO can be easily modified and tuned to its physicochemical properties. Amines, acyl chloride, aldehyde, and polymers are said to be able to modify GO. However, functionalized GO by polydopamine polymer showed a superior modification efficiency for different membranes, as illustrated in Table 5 by several studies. For instance, barrier layers of PDA-f-GO films were formed on the h-PAN support by a vacuum filtration technique [100]. After 2 h of reaction time, the PDA-f-GO composite membrane showed excellent separation performance, with a permeation flux of 2273 g MH, which was 39% higher than the GO composite membrane in the pervaporation experiment. The deposition of PDA-f-rGO film onto the membrane surface can enhance its super-hydrophilic and underwater super-oleophobic properties. This has been demonstrated through developing PDA-rGO film under vacuum filtration onto a mixed cellulose ester (MCE) filter membrane, leading to high separation efficiency for a variety of surfactant stabilized oil-in-water emulsions and excellent anti-fouling properties. Besides that, membranes showed high chemical stability against acidic, concentrated salt, and weak alkaline conditions [108]. The superoleophobicity of the PDA-f-GO-based membrane was also proved by measuring the contact angles of different organic solvents on the prepared rGO-PDA-PFDT membrane, which was almost zero [109]. Moreover, A hollow fiber isotactic polypropylene (iPP) membrane was synthesized successfully by the bio-inspired PDA-f-GO layer via a facile surface modification process, showing excellent recyclability and antifouling ability under oil-water emulsion separation [110]. For increasing dopamine-functionalized GO (GO-PDA) antibacterial properties, zwitterionic polymer PEI has been used in several studies due to its antibacterial activity and excellent binding ability on the membrane surface [104]. When GO and PDA are combined in Tris(hydro-xymethyl)aminomethane hydrochloride (Tris-HCl) buffer, a covalent cross-linking reaction occurs between PEI and the catechol functional groups in GO-PDA. The fabricated GO@PDA/PES NF membrane through the filtration-assisted assembly strategy showed good antifouling ability and structural stability after being grafted by Z-PEI and achieved a permeability of 49.5 LMH/bar with a relatively high rejection of about 100% for Congo Red, 82% for Orange G, and 67% for Methyl Orange at optimal zwitterionic polymer grafting values [102]. Polydopamine has an ability to bind heavy metals due to the existence of amino and catechol functional groups that can additionally enhance the adsorption functionality of GO membrane for heavy metals. According to the prepared graphene oxide-polydopamine-(β-cyclodextrin) GPC membrane, obtained by the dip-coating method assisted by vacuum filtration of β-cyclodextrin (CD)-grafted GO PDA hydrogel onto non-woven fabrics [101], the membrane showed a high rejection percent for methylene blue (MB) molecules (99.2%) and for Pb^2+^ ions adsorption potential reached a maximum value of 101.6 mg g^−1^, due to the abundance of oxygen-containing groups and the presence of β-CD [101]. Another method for improving membrane dye rejection is to intercalate dopamine-functionalized graphene oxide (DGO) nanosheets into 2D nanosheets such as titanium carbide (MXene-Ti3C2Tx) nanosheets, which are vacuum filtered over membranes such as nylon and PVDF membranes [106,112]. Furthermore, some researchers has shown that metal-organic frame-work (MOF) materials such as HKUST-1 and UiO-66 have been broadly involved and employed as modifiers of GO-based membranes that can enhance their functionality in the purification of dye wastewater [105,107]. The MOF materials UiO-66 or HKUST-1 were intercalated into the GO nanosheets under the modification of polydopamine (PDA), in which the prepared PDA/RGO/MOF composite suspension was vacuum filtered onto the cellulose acetate (CA) substrate, which showed an enhancement in membrane hydophilicity and water permeation flux compared to the PDA-RGO membrane [105,106]. The MOF modified membranes maintained a high dye separation performance (99.54% for MB and 87.36% for CR) when using UiO-66 and (99.8% for MB and 89.2% for CR) when using HKUST-1 [105,107]. Instead of depositing PDA-f-GO onto membrane a substrate by vacuum filtration, some studies have used pressurized assisted self-assembly (PAS) to deposit GO-PDA NPs on a PS-30 substrate [82]. In the same study, it has been shown that GO-PDA NPs have high dispersibility in polar and nonpolar solvents compared to the poor dispersibility of GO in some solvents, which causes agglomeration [82]. This may be due to PDA’s hydrophilic functional groups, which aid in the dispersibility and stability of GO NPs. Dopamine-functionalized GO has been used as an intermediate layer through simple immersing coating technique or a modified molecular layer-by-layer (modified mLBL) method to enhance FO-TFC membranes of various support membranes such as PSF, PVDF, and PAN membranes [96,97]. The TFC membrane with the PSF–PDA/GO support layer enhanced water flux without compromising the reverse solute flux (RSF) [97]. The PDA/GO-coated layers reduce substrate surface roughness, allowing the PA layer to develop more easily [96]. Furthermore, PDA-f-GO can increase FO-TFC membrane antibiofouling performance through deposition onto the surface of the rejection active layer, which also improves its smoothness and hydrophilicity [98]. The same technique has been followed in order to obtain a bactericidal and antibiofouling surface for the commercial RO membrane (BW4040 AFR) using GO crosslinked with a thin layer of polydopamine (PDA-f-GO) [111]. Instead of using (PDA-f-GO) for modifying the surface of the membrane support layer as discussed before, it can be injected into or blended with the support polymer matrix via the phase inversion technique due to the high dispersion of rGO-PDA. The prepared PES nanocomposite membranes tested in ultra-low-pressure reverse osmosis (ULPRO) desalination application demonstrated that blending PDA-f-GO with polymer matrix can increase membrane salt rejection up to 99.9% [99]. Moreover, blending PDA-f-GO with the casting solution can greatly enhance the flux, hydrophilicity, pore structure, antifouling properties, and surface roughness of casted membrane more than the pristine membrane or GO-based membrane, as was proved by the fabricated UF membrane of PSF/rGO-PDA mixed matrix membranes (MMMs) [83].

## 6. Membrane’s Modification Based on PDA Nanoparticles Incorporation

PDA nanoparticles can be prepared by a facile technique based on the oxidation and self-polymerization of dopamine spontaneously under basic conditions at room temperature (~25 °C). Under stirring in the presence of air oxygen, DA is dissolved in a mixed solution of DI water, ethanol, and ammonia until the colorless solution turns pale yellow and then brownish black. After that, the prepared particles are centrifuged to separate them, followed by thoroughly rinsing with DI water and drying in the oven [113]. These NPs have been used in many applications due to their exceptional biodegradability, simplicity, adhesiveness, film formability, biocompatibility, and durability. DPA nanoparticles have antioxidant properties too. For instance, it has been used in drug delivery applications [113], imaging of cells and tissues, sensing of target molecules, and antibacterial applications [114,115,116,117,118]. Therefore, over the past few years, PDA NPs have been extensively used in membrane-based separation technology as durable and eco-friendly nanofillers to boost membranes’ efficiency.

Most of the studies have incorporated PDA NPs in membrane modification by blending them with the membrane polymer matrix. For example, a polyethersulfone (PES)-UF membrane has been modified using sulfonated-functionalized polydopamine (SPDA) nanofillers via a non-solvent-induced phase separation process (NIPS) [119]. Using the same technique, a fabricated PVDF membrane has been modified using PDA NPs [120]. The findings of both studies showed that PDA nanofillers have a lot of potential for improving membrane permeability and antifouling capabilities without sacrificing their separation efficiency. These polymer/PDA NPs blend membranes also showed long-term stability in the aqueous environment due to the strong interactions between PDA NPs and polymer chains. In addition, PDA NPs have exhibited good performance in enhancing TFC membranes under the FO treating process [121,122]. Significant ICP reduction and structural stability improvement of TFC electrospun polyacrylonitrile nanofiber membrane were observed after depositing PDA NPs as an interlayer onto the membrane substrate [121]. This also increased the membrane substrate hydrophilicity and the adhesion strength between the selective layer and the substrate. Another modification method is the incorporation of PDA NPs into interfacial polymerization, which can form a stable chemical cross-linking structure with the TMC organic phase during the IP process [123]. These NPs could also establish more interfacial channels with polyamide macromolecules, providing more pathways for water molecules passing through the membrane. Furthermore, PDA NPs can provide new chances for enhancing membranes by creating hybrid nanoparticles with other inorganic NPs such as Ag-PDA NPs. These hybrid nanoparticles showed promising results in modifying PES matrix membrane performance and antibacterial properties [124]. On the other hand, PDA NPs have poor thermal stability. Thus, functionalizing PDA NPs by high-thermal-stability methoxy polyethylene glycol amine (mPEG-NH_2_) showed an ability to construct antifouling melt blend composite membranes [125]. It can be said that PAD NPs with their multifunctional properties have shown promising prospects in enhancing different water purification membranes.

## 7. Conclusions and Perspectives

Polydopamine with its unique properties has confirmed its ability to decrease nanomaterial agglomeration and leaching from membranes and improve their interfacial interactions and poor compatibility with polymeric membranes. PDA can be incorporated by different methods using various types of organic and inorganic nanomaterials for enhancing the performance of various water purification membranes such as UF, MF, NF, RO, and FO membranes. This approach has been extended to the surface modification of nanofillers. PDA-f-NPs demonstrated considerable progress in this field. According to the membrane modification and fabrication process, PDA-f-NPs have been used to modify the surfaces of membrane support and rejection layers by simple coating and deposition. They have also been used as an interlayer between membrane layers, incorporated into membrane polymer matrix via the phase inversion method and finally introduced into the PA layer through the interfacial polymerization (IP) method. PDA-f-NPs and PDA NPs both showed impressive advances in membrane surface modification and performance. Among various NPs, cross-linking of PDA-f-GO particles has good prospects for future investigation. Nonetheless, more research progress in DA polymerization mechanism, composition, and the formation kinetics of the PDA adhesive layers at the surface of the NPs materials is still needed. Moreover, when it comes to membrane fouling resistance testing, most laboratory research relies on single compounds such as BSA, HA, and other model foulants. However, multi-pollutant removal from surface water and industrial wastewater treatment applications are still rarely reported. Moving applications from the lab to the full scale is still difficult due to a few major hinderances, such as the capital and operational costs, fouling control, and choice of the NPs additives based on large scale process treatment, so the membrane modification procedures must be scaled up and implemented, utilizing actual process feed streams.

## Figures and Tables

**Table 1:** Studies based on two-step and one-step modification methods for UF, NF, and MF membranes.

Membrane Type	Tested on	Filler	NPs Concentration	Methods	Solute/Application	Parameters Achieved	References
Poly (ether imide) (PEI)-UF	Dead end filtration setup	PEI/PDA/Ag NPs	0.005 M and 0.01 M AgNO_3_ solution	Two-step modifications	BSA, HA, and Oil	J_w_ = (97.2 LMH) Hydraulic resistance (13.8 kPa/LMH) Rejection (>97%) Flux recovery ratio FRR (>95%)	[13]
Polyethersulfone (PES)-UF	Cross-flow filtration setup.	PES/PDA/TiO_2_ NPs	0.1 and 0.5 (*w*/*v*%) of TiO_2_	Two-step modifications	BSA	FRR = 32% BSA rejection = 84% 50% flux reduction	[20]
Polyethersulfone (PES) membrane surface	Protein adsorption and bacteria experiments.	PDA-(PEI-SBMA)-AgNPs Polyethyleneimine-graft-sulfobetaine methacrylate	0.1 M of AgNO_3_ solution	Co-deposition and two-step modifications.	Protein and bacteria	High antibacterial properties.	[22]
PPMM polypropylene- MF	Dead-end filtration equipment	PDA-PEI- TiO_2_	2.5 × 10^−5^ M of Ti-BALDH and 0.025 M of NH_3_. H_2_O	Co-deposition and two-step modifications.	BSA and Lys	FRR = 82% for BSA solution. FRR = 86% for Lys solution. Relative flux reduction (RFR) = 31 for BSA solution RFR = 26% for Lys solution.	[23]
Commercial Polyacrylonitrile PAN-UF sheet membrane-150 kDa	Cross-flow filtration setup.	PDA-PEI-CuSO_4_/H_2_O_2_	8.3 mM CuSO_4_ and 32.6 mM H_2_O_2_	Rapid Co-deposition	Salts (Na_2_SO_4_, MgCl_2_) Dyes	Water permeability (26.2 LMH/bar) Dyes rejection > 90%	[24]
PA-TFC-NF NFX-TFC membranes (NF)	Dead-end cell (High Pressure Stirred Cell Kit).	PDA–TiO_2_ PDA–ZnO PDA–TiO_2_:ZnO	0.01, 0.02, 0.03, 0.05, 0.005, 0.007 and 0.015 wt% of TiO_2_ and ZnO	Two-step deposition and co-deposition.	Salts: NaCl and Mg_S_O_4_Bacillus Subtilis as model bacteria	Water permeability = 6.8, 7.7 and 7.8 LMH/bar for TiO_2_ co-deposition. Water permeability = 6.8, 6.2 and 5.9 LMH/bar for TiO_2_ two-step coating. MgSO4 rejection ~95%	[14]
Commercial Polyacrylonitrile PAN-UF sheet membrane −75 kDa	Cross-flow filtration setup.	PDA-CuNPs	25 mL and 40 mL of CuNPs solution	Two-step deposition and co-deposition.	Dyes. Bacteria Salts.	Textile dyes rejection >99% Water permeability = 18.6 LMH/bar for two-step coating. Water permeability = 25.5 LMH/bar for co-deposition of PDA and CuNPs	[26].
Commercial Polyacrylonitrile PAN-UF membrane- ranging from 10 to 30 kDa	Cross-flow filtration setup.	PDA-PEI-SiO_2_ NPs	0–2 mg/mL of SiO_2_ NPs	Co-deposition	Various salts: NaCl, CaCl_2_, MgCl_2_, MgSO_4_, Na_2_SO_4_	J_w_ = 32 LMH Bivalent cations whereas rejection = 90% Monovalent cations < 30%	[27]
Commercial Polyacrylonitrile PAN-UF-50 kDa	Cross-flow filtration setup.	PDA-PEI-GNPs (electropositive gold NPs)	Same designed concentration of GNPs.	Co-deposition	Metal salts (ZnCl_2_, BaCl_2_, NiCl_2_, and CdCl_2_) Salts (MgCl_2_) and NaCl	J_w_ = 240 LMH MgCl_2_ rejection > 90% Na_2_SO_4_ rejection < 30% 50% reduction of bacteria	[28]
Commercial Polyacrylonitrile PAN-UF membrane- 100 kDa	Dead-end stirred cell filtration apparatus	POSS (NPs)-PDA	12 mg of POSS solution	Co-deposition	Dye solution and salt solution	Water permeability 1099 LMH/MPa. Dye rejection (>90%) Salt permeation (>90%)	[32]
Hydrolyzed Polyacrylonitrile (HPAN-UF) membrane	Home-made cross-flow filtration cell.	g-C_3_N_4_ nanosheets -PDA/polyethylenimine (PEI)	0–0.005–0.01–0.02–0.04% of C_3_N_4_ nanosheets suspensions.	Co-deposition	Dye and salt	Water permeability = 28.4 LMH/bar. Dyes rejection >99.3%. Low salt rejection: 2.9% rejection of NaCl and 7.6% for Na_2_SO_4_	[31]
SiO_2_/PVA electrospun nanofiber membrane	Suction filter device	Reduced Pd NPs decorated Polydopamine	150 mg of PdCl_2_	Co-deposition	Organic compounds oils and dyes (kerosene, hexane, petroleum ether, chloroform and toluene)	J_w_ = 8000 LMH Removal effeciency of oils and organic chemistry 99.9%. Degradation effeciency of Dyes: 99%.	[29]
polyvinylidene fluoride (PVDF) ultrafiltration (UF) membrane	Dead-end flow stirred cell	Halloysite nanotubes (HNTs)-3-aminopropyltriethoxysilane (ABTES)-PDA	120 mg of HNTs	Co-deposition	BSA	J_w_ = 291.9 LMH Rejection of BSA = 92%	[30]
Commercial PAN-100,000 Da	Dead end filtration setup	PA/PDA-COF (covalent organic framework nanosheets)/PAN	0–0.35 g/L	Co-deposition	Salt and dye	Water permeability = 207.07 LMH/MPa Salt rejection > 90% Dyes rection 92.8–99.9%	[33]
Cellulose acetate (CA) membrane	Vacuum filtration	Hal@MXene NPs -PDA	2 mg Mxene	Co-deposition via vacuum filtration	Oil-water emulsion	Water permeability = 5036.2 LMH/bar Rejection of oil > 99.8%	[34]

**Table 2:** Studies based on different modifications methods using PDA-f-NPs for UF and NF membranes.

Membrane Type	Tested On	Filler	NPs Concentration	Methods of PDA-f-NPs Deposition	Solute/Application	Parameters Achieved	References
Commercial PES membranes-UF	Dead-end Filtration cell	PDA-f-TiO_2_	0.05 wt% of TiO_2_	One-step dip coating	BSA	J_w_ = 962 LMH FRR = 97% Fouling reversibility = 98.62%	[16]
Laboratory made PES/UF membranes via casting	Crossflow filtration cell	MWCNTs coated by metal/metal oxide (Ag, Al_2_O_3_, Fe_2_O_3_ and TiO_2_) then coated with a PDA layer to produce HNS.	50 mg of each HNS were added to DA solution.	Vacuum filtration deposition method for depositing PDA-coated HNS onto membrane substrate. TFN membranes were fabricated via interfacial polymerization	Salts (NaCl, Na_2_SO_4_ and MgSO_4_)	J_w_ = 10.5 LMH Salt rejection = 97.15–99.44%	[49]
Nanofiltration membranes with a polyamide selective layer and a poly (ether sulfone) (PES) support layer	Crossflow Filtration cell	Cu-MOF NPs-PDA	1 wt% of Cu-MOF NPs were added to DA solution.	(Dip-coating) and dynamic (filtration-assisted)	Dyes (Methylene blue and methyl orange)	Dyes rejection = 98%. 43 and 37 LMH	[50]
PSf-based hybrid membranes	Crossflow Filtration cell	TiO_2_-PDA nanohybrid	Prepared TiO_2_-PAD particles	Phase inversion method	BSA	J_w_ = 428 LMH FRR = 72%	[53]
PVDF-UF	-	PDA-TiO_2_	1 wt% of PDA-coated TiO_2_	Phase inversion method	-	Flyx increased by 35.7%.	[54]
PSf-UF	Crossflow Filtration and Dead-end Filtration.	MWCNTs-PDA	0.1–0.5 wt.% MWCNTs-PDA	Phase inversion method	Organic solutions BSA solution	J_w_ = 81.27 LMH R% of BSA = 99.88%	[55]
PES	Dead-end Filtration cell.	PDA@ZnFe_2_O_4_ NPs	2 wt% and 4 wt% of PDA@ZnFe_2_O_4_ NPs	NIPS method (casting)	Humic acid Oil-water separation	J_w_ = 687 LMH R% of HA = 94% R% of oil = 96% FRR for HA = 94% FRR = for oil = 82.5%	[56]
PAN-UF	Dead end Filtration cell.	polydopamine modified silica nanoparticles (SiO_2_-DOPA)	5–10–15% of (SiO_2_-DOPA)	Phase inversion process.	rejection of BSA protein and dye molecules	FRR = 75%	[51]
Cellulose acetate (CA)	Crossflow filtration setup	P(DA-SBMA) nanoparticles	0.05–0.1–0.2 and 0.3 wt%	Wet-phase inversion	oil-in-water emulsions	Jw = 583.64 LMH FRR = 8.85% Reversible fouling = 11.10%	[57]
PSF membrane fabricated by Nonsolvent induced phase separation (NIPS) method	Home-made Crossflow filtration apparatus	PDA-zeolitic imidazolate framework-8 (ZIF-8) NPs	0.01 wt% of PDA-(ZIF-8) NPs	Incorporated into PA layer within aqueous phase during interfacial polymerization	Salts: (NaCl) (Na_2_SO_4_) rhodamine B	J_w_ = 4.81 LMH R% of SO_4_^2-^	[59]
PSf fabricated via casting	Crossflow Filtration cell.	PSf-PIP/PDA-SiNPs-TMC	PDA-SiNPs/TMC in g/g: 0.05–0.15–0.35–0.55–0.75 and 0.95	PDA-SiNPs into TMC solution (interfacial polymerization)	Bovine serum albumin (BSA) NaHSO_3_, HCl, NaOH, Na_2_SO_4_, Mg_S_O_4_, MgCl_2_, and NaCl. Concentrated ammonia water	J_w_ = 80 LMH R% of Na_2_SO_4_ = 97%; R% of MgSO_4_ = 94%; R% of MgCl_2_ = 68%; R% of NaCl = 35%.	[60]
Polysulfone (PSf) support membranes via casting	Crossflow Filtration cell.	Poly (dopamine-sulfobetaine methacrylate) [P(DA-SBMA)] nanoparticles	P(DA-SBMA)/TMC in g/g: 0.05–0.15–0.35–0.55–0.75 and 0.95	P(DA-SBMA) NPs were dispersed in the TMC phase during interfacial polymerization (IP)	salt rejections Na_2_SO_4_, MgSO_4_, MgCl_2_, and NaCl. Bovine serum albumin (BSA)	J_w_ = 73.11 LMH R% of Na_2_SO = 98% R% of MgSO_4_ = 95%, R% of MgCl_2_ = 54% R% of NaCl = 42%	[58]
Commercial polysulfone (PSf) ultrafiltration membrane (20 kDa)	Self-made Cross flow equipment	ZIF-8@PDA	0.01–0.02–0.03–0.04 wt% of ZIF-8@PDA NPs	ZIF-8@PDA nanoparticles were dispersed in the TMC phase during interfacial polymerization (IP)	NaCl solution, BSA and lysozyme LZM solutions	Water permeability = 3.74 LMH/bar 43.8% higher than control membrane. R% of chlorine = 98.68%	[61]
Commercial polyether sulfone (PES) membrane	Dead-end Filtration cell	PDA-coated ZIF-8 NPs	5–10–20–40 wt % of PDA-coated ZIF-8 NPs based on the weight of PA selective layer.	PDA-f-ZIF-8 NPs dispersed in the aqueous solution of MPD	NaCl, Na_2_SO_4_, HA	Water permeability = 11.4 LMH R% of NaCl = 45.4 % R% of Na_2_SO_4_ = 95.1% FRR% = 94.4%	[48]

**Table 3:** Studies of FO membranes modification by PDA freestanding and PDA with NPs-based modifications.

Membrane Type	Filler	Method	Solute/Application	Parameters Achieved	References
**Commercial BW30 and SW30-XLE** **Reverse osmosis membranes made of PSu supported by a PET nonwoven**	Isopropanol (IPA) -PDA	Coating	NaCl	Four-to-six-fold increase in FO water flux. Chloride ion rejection = 80–90%	[63]
**PSF membrane via wet phase inversion method**	PDA/(MPD-TMC)	Coating PDA as Intermediate layer	NaCl	J_w_ = 24 LMH RSF 1.75 gMH	[64]
**Polyketone (PK)-based TFC membrane-FO via induced phase separation (NIPS) method**	Poly(2-methacryloyloxyethyl phosphorylcholine-co-2-amino-ethyl methacrylate hydrochloride) (MPC-co-AEMA)-PDA	Modified by Co-deposition (single-step simultaneous deposition) over rejection layer.	Oil and bovine serum albumin (BSA).	R% = 95.2% J_w_ = 23.7 (LMH) J_s_ = 4.9 (g MH)	[68]
**CA membrane via non-solvent induced phase separation.**	CA- PVA-PDA	PVA and PDA by Surface coating technology	NaCl	J_w_ = 16.72 LMH J_s_ = 0.14 mMH Salt R% = 96.4%	[67]
**Mixed cellulose ester (MCE) substrate**	TMC/MPD-DA/MCE TMC/DA/TMC	Incorporated into PA layer- within MPD aqueous phase during interfacial polymerization	NaCl	J_w_: (50 LMH, J_s_: 8.19 gMH NaCl rejection > 92 % at 2 bar.	[69]
**PSF via phase inversion method**	PDA-LDHs (Layered double hydroxides)	Coating TFC membrane by PDA as Intermediate layer Then immersed in the LDHs suspension for 1 h	Sodium alginate NaCl CaCl_2_	FO mode J_w_ = 9.93 LMH and 9.99 LMH J_s_ = 4.9 gMH and 4.8 gMH	[70]
**PVC support membranes via Phase inversion**	TFC- PDA coated over PVC membrane	Coating (1–3 h) PDA onto PVC surface as intermediate layer	NaCl	J_w_ = 18.9 LMH (FO mode) and 47.5 LMH (PRO mode) J_s_ =3.35 gMH (FO mode)	[65]
**polyethylene (PE) support,**	PDA over PE-TFC	Simple dip coating (8 h) in PDA as intermediate layer	NaCl	FO-Mode J_w_ = 53.0 LMH J_s_ = 14.82 gMH	[66]
**TFC (consists of a polyamide rejection layer and a porous supporting layer embedded on a polyester mesh**	TFC-PDA	Surface Coating only the rejection layer PA exposed to the coating solution	NaCl	J_w_ = 9 LMH at FO mode Reverse solute diffusion = 0.90 g/L at FO mode J_w_ = 16.5 LMH at PRO mode reverse solute diffusion = 0.82 g/L at PRO mode	[71]
**PSF via casting**	TMC/DA-MPD/PSF	Interfacial polymerization	NaCl Humic acid	J_w_: (15.09 LMH) at FO mode. J_s_: (32.77 mmol m^−2^ h^−1^) at FO mode	[72]
**Porous polysulfone membrane substrate PSf-PVP via casting**	DA/TMC TFC	The PSf substrate was first immersed in DA solution then dipped into TMC solution. IP reaction occurred between DA and TMC	MgCl_2_ solution Chlorine resistance (NaClO solution)	J_w_ = 6.55 LMH, J_s_ =1.1 g/L.	[73]
**Double-layer polyacrylonitrile (PAN) casted**	polydopamine/metal organic framework	Rapid co-deposition of polydopamine (PDA) and MPD. MOF-801 (0.005–0.01–0.02 wt%) dispersed in a 0.1 wt% TMC/n-hexane solution then poured over PDA/MPD membrane via IP.	Salt Heavy metal ion rejection (Cd^2+^, Ni^2+^, Pb^2+^)	Salt rejection 87.94%, 93.5%, and 85.7% (94~99.2% for Ni^2+^, Cd^2+^, and Pb^2+^ removal rate)	[15]
**Commercial polyethersulfone (PES)-microfiltration (MF) membrane**	PDA-single-walled carbon nanotubes (SWCNTs)	Vacuum filtration + spraying Amount of PAD-SWCNTs dispersion 0–3–9–15–21 mL.	NaCl, Bovine serum albumin (BSA)	J_w_ of 35.7 LMH at PRO mode J_s_ of 1.42 gMH at PRO mode BSA R% = 98%	[74]
**Commercial Polyethersulfone ultrafiltration membrane**	(ZIF-8@PDA) in the poly (ethyleneimine)/1,3,5-benzenetricarboxylic acid chloride (PEI/TMC) crosslinked matrix	Deposition of (0–0.025–0.05 and 0.1 wt% of ZIF-8@PDA) in the poly (ethyleneimine) onto membrane substrate. Followed by 1,3,5-benzenetricarboxylic acid chloride (PEI/TMC) crosslinked matrix Via IP	MgCl_2_ solution heavy metal wastewater (Cu^2+^, and Ni^2+^ and Pb^2+^)	J_w_ of 20.8 LMH J_s_ = 5.2 gMH Heavy metal ions rejection (>96%)	[75]

**Table 4:** Studies based on incorporation techniques of GO and PDA into different membranes modification.

Membrane Type	Tested on	Filler	GO NPs Concentration	Modification Technique	Target Solute (Applications)	Methods	Parameters Achieved	References
**Commercial Mixed cellulose ester membrane** **(CTA-ES)**	Pressurized filtration tests and FO process system	Silver nanoparticle (nAg)@polydopamine (PDA)-rGO membrane	0.006 mg/mL GO aqueous solution	Surface modification (onto substrate surface)	Sodium chloride (NaCl) Pseudomonas aeruginosa PAO1 was used as a model microorganism for (biofouling propensity)	(Vacuum-filtered deposition of GO+ Dipping into DA solution and then deposit silver nitrate solution)	R% of salt nAg@pDA-rGO (65.6%) and pDA-rGO (59.5%). J_w_: pDA-rGO (34.0 LMH). J_w_: nAg@pDA-rGO (28.9 LMH). J_s_: 1 mol/m^2^/h for pDA-rGO J_s_: 0.85 mol/m^2^/h for nAg@pDA-rGO	[94]
**Commercial Mixed cellulose ester (MCE) membrane**	FO system	polydopamine/R-graphene oxide (PDA-rGO)	0.006 mg/mL GO aqueous solution	Surface modification (onto substrate surface)	sodium chloride (NaCl)	Vacuum filtration deposition of GO + dipping in dopamine solution.	J_s_: 0.04 mol/m^2^h J_w_: 29.8–36.18 LMH R% of salt = 92%.	[95]
**Commercial polysulfone (PS-UF)**	NF Experiment	PSF/PDA/TMC/GO	0.5 g/L of GO solution	PDA as intermediate layer. GO was grafted onto PA layer	organic dyes and salt solutions (methyl blue, Congo red, acid fuchsin, crystal violet, methyl orange) NaCl, Na_2_SO_4_, and Na_3_ PO_4_	LBL self-assembly method (immersion)	R% of MB = 78% Permeation flux of MB = 70 kgm^−2^h^−1^ R% of PO_4_^−3^ = 92% Permeation flux PO_4_^3−^ = 120 kgm^−2^h^−1^ FRR = 90%	[91]
**PES membrane via phase separation method**	NF Filtration system	GO-PDA/PES	5 mg/L of GO	Surface modification (onto substrate surface)	Dyes	PDA layer via Coating + filtration-assisted assembly strategy for depositing GO.	Water permeability = 85 LMH/bar R% of Dyes = 95%, 100%	[84].
**poly(arylene ether nitrile) PEN nanofibrous by electrospinning**	Dead-end flow filtration experimental device connected with a solution reservoir at constant pressure of 0.1 MPa	PEN/GO-PDA	25 µ/mL of GO	Surface modification (onto substrate surface)	Dyes (Direct Blue 14, Direct Red 28, Direct Yellow 4, and Methylene Blue)	GO skin layer formed by Vacuum filtration. Followed by Immersing PEN/GO nanofibrous into dopamine solution	Permeate flux = 99.7 LMH R% of Direct Blue 14 = 99.8%	[88]
**Commercial NF90 membrane**	NF Experiment	PDA-GO	50 mg/L of GO solution.	Printed on the membrane surface.	NaCl	Inkjet printing	Water permeability = 11.63 LMH/bar R% of salt = 92.42%	[93]
**Polyvinylidene fluoride (PVDF via phase inversion** **method**	Vacuum filter apparatus	PVDF /RGO@SiO2/PDA nanohybrid membranes	2 mg of GO/(0.67, 1.34, 2, and 2.67) mg of SiO_2_	Surface modification (onto substrate surface)	Oil water emulsion dye wastewater (MB)	Vacuum-assisted filtration self-assembly process for depositing RGO@SiO2 film onto membrane surface. Then RGO@SiO_2_ membrane soaked into DA solution	Water permeability = 475.5 LMH/bar R% of MB = 98% FRR% = 87.2%	[89]
**Electrospun of poly (arylene ether nitrile) PEN nanofibrous mats (supporting layer)**	Oil/water separationvacuum filter apparatus	Poly (arylene ether nitrile) (PEN)/HNTs@GO-PDA nanofibrous composite membranes	50 µ/ML of GO. (0, 25, 50, 100 and 150) µ/mL of PDA modified HNTs	Surface modification (onto substrate surface)	Oil/water emulsion	(HNTs intercalated GO hybrids were assembled onto porous PEN supporting layer by Vacuum filtration deposition. Followed by crosslinking of dopamine.	J_w_ = 1130.56 LMH R% > 99%	[87]
**Mixed cellulose ester membrane (MCEM)**	Vacuum filter apparatus	GO/PDA/MCEM	100 mg/L of GO suspension.	Surface modification (onto substrate surface)	Oil	PDA deposited by oscillation incubator for 24 h. Followed by GO deposition by Vacuum filtration	Permeate flux = 146 LMH/bar R% of oil = 96%	[85]
**Porous alumina** **α-Al_2_O_3_ supports.**	Tested for Seawater desalination at 30~90 °C by pervaporation	GO-PDA	0.01–1 mg/mL of GO suspension.	Surface modification onto Al_2_O_3_ support surface	Sea salt	Vacuum filtration of GO onto PDA-Al_2_O_3_ supports	J_w_ = 48.4 LMH ’R% of oi; >99.7%	[86]
**Commercial Polysulfone (PSf)-UF**	Crossflow Filtration cell-UF	PDA/aGO(activated)	0.1 g/L GO solution	Surface modification onto UF substrate surface	Sodium alginate (SA)	Coating of PDA and Grafting of aGO.	Water permeability = 830 LMH/bar J_w_ = 135 LMH Flux increased by 20% Fouling rate reduced by 63%	[92]
**n-poly-(ethylene terephthalate) PET-UF**	-	rGO-PDA-PET	-	Surface modification (onto substrate surface)	-	Drop-casting methodPET substrates were immersed in an aqueous solution of dopamine. GO dispersion was drop casted onto the polydopamine-modified PET substrates.	-	[90]

**Table 5:** Studies based on different membrane’s modifications techniques based on dopamine-functionalized GO nanoparticles.

Membrane Type	Tested on	Filler	Deposition Time of PDA-f-GO Concentration of DA and GO NPs	Modification Technique of PDA-f-GO Layer	Target Solute (Applications)	Methods	Parameters Achieved	References
**Five substrates:** **Hydrophilic poly (vinylidene fluoride) membrane (PVDF)** **Highly hydrophilic PVDF** **Nonwoven PAN** **Freestanding PAN** **Titania-coated carbon nanotube (TCNT) on the nonwoven PAN substrate**	FO system setup	PDA-f-GO then polyethylenimine/poly (acrylic acid) (PEI/PAA) layers and subsequent PA layer formation.	4 h 1 g DA and 20 mg GO Added into Tris solution	As an intermediate layer	Sodium chloride (NaCl)	PA formed via layer-by-layer method. Coating of PDA-GO as interlayer (soaking membrane in PDA-GO solution for 4 h)	J_w_ = 6.75 (LMH) J_s_ = 1.7 (gMH) By mLBL3 for the nonwoven PAN	[96]
**polysulfone (PSf) support via phase inversion**	FO system setup	polydopamine/graphene oxide (PDA/GO) interlayer-PA	(1–5 h) 0.1 g DA and 5 mL of the GO–DI water mixture Added into Tris solution	As an intermediate layer	Sodium chloride (NaCl) PEG	PDA/GO layer formed via (Immersing, coating) of PSF membrane. PA layer formed through Interfacial polymerization.	J_w_ = 24.296 LMH J_s_ = 3.818 gMH	[97]
**Flat sheets TFC FO membrane (HTI, OsMem™ TFC Membrane**	FO system setup	PDA-GO	Various GO concentration and various deposition time	Grafting onto PA rejection layer	NaCl	Coating and shaking	J_w_ = 13.63 LMH J_s_: 0.68 mg/min	[98]
**polyethersulfone (PES) membrane**	ultra-low pressure reverse osmosis (ULPRO)	dopamine-stabilized graphene-based (xGnP-DA)	Not available	Blending with polymer matrix	NaCl and Synthetic Seawater Solutions	Phase inversion. Casting. PES + PVP + NMP + (GO + DA)	J_w_ = 19LMH at 8 bar FRR% = 99.9% R% = 99.95%	[99]
**Polysulfone membrane**	UF system setup	rGO-PDA	Not available	Blending with polymer matrix	BSA Bovine serum albumin HA Humic acidSO, ORII, MB and DR80 dyes	Phase inversion technique.	Water permeability = 326.5 LMH/bar R% of BSA = 100% R% of MB = 87% FRR% of BSA = 80.4 % FRR% of HA = 99.4%	[83]
**Hydrolyzed commercial polyacrylonitrile** **(h-PAN)**	UF system setup	PDA-GO	75 mg DA 75 mg GO Added into Tris solution	Surface modification onto substrate surface	Ethanol–H_2_O mixture isopropyl alcohol–H_2_O mixture	simple vacuum filtration method.	Permeation flux = 2273 g m^−2^ h^−1^	[100]
**Non-woven fabrics (purchased from Paper Group Company).**	Dead end filtration cell	GO-PDA- β-cyclodextrin (CD)	16 mg GO into 1.8 mL DIW. Then 0.2 mL of DA solution (4 mg mL^−1^) added into the GO solution with the pH = 11	Surface modification onto substrate surface	Organic molecules (methylene blue) Trace heavy metals (Pb^2+^).	Drop-coating combined with vacuum filtration.	J_w_ = 12 LMH R% of MB = 99.2 %	[101]
**PES membrane via (phase inversion)**	NF system setup	Zm-PEI-GO@PDA/PES	25 mg DA 50 mg GO powder Added into Tris solution	Surface modification onto substrate surface	Organic dyes	GO@PDA/PES via Filtration assisted assembly strategy.Zwitterionic polymer was grafted on the surface of PDA crosslinked GO membrane.	Water permeability = 49.5 LMH/bar R% of Congo Red = 100%, R% of Orange G = 82% R% of Methyl Orange = 67%	[102]
**Commercial Cellulose acetate (CA) substrate**	Vacuum extraction filter	PDA/RGO/UiO-66	25 mL GO solution 0.1 g DA	Surface modification onto substrate surface	dye wastewater.	vacuum-assisted filtration self-assembly method.	J_w_ = 167.14 LMH R% of MB = 99.54% R% of Congo Red = 87.36%	[103]
**CA support**	shake flask method	GO-PDA. GO-PDA-PEI	Not available	GO-PDA-PEI membrane was peeled off from the CA support.	Bacterial cells (*S. aureus* and *E. coli*)	vacuum-assisted filtration self-assembly	Antibacterial efficiency > 99%	[104]
**Cellulose acetate membrane support layer**	vacuum suction device	PDA/RGO/HKUST-1 metal-organic frameworks (HKUST-1)	125 mg GO 50 mg DA Various (HKUST-1) concentrations.	Surface modification onto substrate surface	dye wastewater. (Methylene blue and Congo red)	vacuum filtration.	J_w_ = 184.71 LMH R% of MB = 99.8% R% of Congo Red = 89.2%,	[105]
**Commercial PS30**	UF system setup	GO-PDA NPs	100 mg GO powder and 200 mg DA added into Tris solution	Surface modification onto substrate surface	-	pressure-assisted self-assembly technique (PAS)	-	[82]
**Commercial Hydrophilic polyvinylidene fluoride (PVDF) membranes**	Molecular dynamics (MD) simulations	Dopamine-functionalized graphene oxide (DGO) -MXene(Ti_3_C_2_Tx)	0.4 g GO NSs dispersed in DI and 0.02 g DA added into Tris solution	Surface modification onto substrate surface	Dye and salts mixed solution (NaCl, MgSO_4_)	vacuum filtration deposition	J_w_ = 63.5 LMH Dyes rejection 98.1% and 96.1%	[106]
**Commercial Cellulose acetate (CA) membrane**	NF system setup	PDA/RGO/halloysite nanotubes (HNTs)	Not available	Surface modification onto substrate surface	Oil water emulsion, Dyes and heavy metals	Vacuum filtration deposition	Permeate flux = 23.53–60.32 LMH R% = 99% FRR% = 82.27%	[107]
**Mixed cellulose ester (MCE) filter membrane**	Separation device (Vacuum filtration system)	PDA-rGO	Not available	Surface modification onto substrate surface	Oil	Vacuum filtration	Separation efficiency = 99.6%	[108]
**rGO-PDA-1H,1H,2H,2H-perfluorodecanethiol** **PFDT membrane**	Separation device (Vacuum filtration system)	rGO-PDA-PFDT	3 mg DA and 3 mg GO	-	Oil (organic solvents)	Vacuum filtration through a Whatman filter paper.	=	[109]
**isotactic polypropylene(iPP) hollow fiber membrane via thermally induced** **phase separation (TIPS) process**	Membrane filtration system	iPP@PDA@GO membrane	200 mg DA, 200 mg GO and 200 mg APTES	Membrane immersed into PDA + GO + APTES solution	Oil	Immersing coating	Oil-water permeation = 188 LMH in 0.1 MPa Oil rejection (>99%)	[110]
**A commercial RO membrane (BW4040 AFR)**	RO lab scale	GO-PDA	GO powder (50 mg) a dopamine solution (300 mL) contains dopamine hydrochloride (2%) added into Tris solution	Onto (top of) the active layer	NaCl	Coating single-pot technique	3.8% decline in the flux value. R% of NaCl > 97%	[111]
**Nylon membrane**	Filtration device	RGO/PDA/MXene (titanium carbide)	100 mg of dopamine hydrochloride. 150 mL of graphite oxide solution—150 mL of a certain amount of MXene solution all added into Tris solution.	Surface modification onto substrate. surface	Oil and chemical dyes	(Vacuum filtration deposition method) RGO/PDA/MXene solution filtered on a dopamine-impregnated nylon membrane	Permeability = 174.16 LMH/bar Dye rejection 95%	[112]
